# Spike bursting in a dragonfly target-detecting neuron

**DOI:** 10.1038/s41598-021-83559-5

**Published:** 2021-02-17

**Authors:** Joseph M. Fabian, Steven D. Wiederman

**Affiliations:** 1 Centre for Neuroscience, Flinders University, Adelaide, SA Australia; 2 Adelaide Medical School, The University of Adelaide, Adelaide, SA 5005 Australia

**Keywords:** Physiology, Neuroscience, Sensory processing, Visual system

## Abstract

Dragonflies visually detect prey and conspecifics, rapidly pursuing these targets via acrobatic flights. Over many decades, studies have investigated the elaborate neuronal circuits proposed to underlie this rapid behaviour. A subset of dragonfly visual neurons exhibit exquisite tuning to small, moving targets even when presented in cluttered backgrounds. In prior work, these neuronal responses were quantified by computing the rate of spikes fired during an analysis window of interest. However, neuronal systems can utilize a variety of neuronal coding principles to signal information, so a spike train’s information content is not necessarily encapsulated by spike rate alone. One example of this is burst coding, where neurons fire rapid bursts of spikes, followed by a period of inactivity. Here we show that the most studied target-detecting neuron in dragonflies, CSTMD1, responds to moving targets with a series of spike bursts. This spiking activity differs from those in other identified visual neurons in the dragonfly, indicative of different physiological mechanisms underlying CSTMD1’s spike generation. Burst codes present several advantages and disadvantages compared to other coding approaches. We propose functional implications of CSTMD1’s burst coding activity and show that spike bursts enhance the robustness of target-evoked responses.

## Introduction

Sensory neurons respond to salient stimuli by generating electrical signals, often in the form of impulses called spikes (action potentials). Neurons encode information about a stimulus within the pattern of spikes over time, and this can be achieved with a variety of different codes. Identifying this code is critical for interpreting neuronal responses, but it is difficult to identify which codes individual neurons are utilising. However, the statistics of spike activity can reveal patterns consistent with specific coding strategies. One representation is spike burst coding, where information is conveyed by rapid bursts of spiking activity, followed by a period of inactivity. This common strategy in the mammalian brain conveys multiple coding advantages^1,2^, however there are no identified examples of spike burst coding in the dragonfly, and only one example in the fly^3^.

Visual neurons in flying insects have been investigated for over 60 years. Dragonflies are aerial predators, which excel at hunting small moving prey and chasing conspecifics during acrobatic pursuits. A subset of neurons in their lobula, the Small Target Motion Detectors (STMDs) are sensitive to the movement of small objects, and likely underlie the detection and tracking of targets during pursuits^4^. Most published work on dragonfly STMDs comes from a particularly interesting neuron, the Centrifugal Small Target Motion Detector neuron (CSTMD1)^5–13^. This neuron possesses remarkably complex response properties including predictive gain modulation^6,7,10,11^ and selective attention^8,12^, proposed to underlie their interception of prey even amidst a swarm. Like many other investigations of neuronal activity, previous descriptions of CSTMD1 quantify response strength by counting spike rates over a temporal window of interest. Here we present additional analysis of CSTMD1’s spiking characteristics and contrast them with other visual neurons in the dragonfly brain. In doing so, we observe the presence of burst coding in CSTMD1’s responses to small moving targets and demonstrate that these bursts represent a robust signal with minimal processing latency.

## Materials and methods

### Electrophysiology

We recorded from 20 immobilised male wild-caught adult Hemicordulia dragonflies, gathered from the Adelaide Botanic Gardens. Animals were immobilised with a 1:1 beeswax–rosin mixture (Colophony Kolophonium, Fluka Analytical), and fixed to an articulating magnetic stand. We tilted the animal’s head forward and dissected a small hole on the posterior surface, directly over the left lobula. We pulled aluminosilicate glass micropipettes on a Sutter Instruments P-97, which were backfilled with 2 mol/L KCl solution (AnalaR), with a typical electrical resistance of 50–150 MΩ. We stepped electrodes into the left proximal lobula complex with a piezoelectric stepper (Marzhauser-Wetzlar PM-10) until a neuron was impaled. Animals were presented with a series of visual stimuli for classification, which included small drifting targets, moving bars, moving gratings and textured patterns. The lobula contains many STMD neurons, but three have been well-characterised; the centrifugal STMD, CSTMD1, and two binocular STMD neurons, BSTMD1 and BSTMD2^7,13^. Here we analyse recordings from these STMD neurons, as well as an ‘optic flow’ Lobula Tangential Cell^14^. Each STMD neuron is identified by its small target selectivity, characteristic receptive field and action potential waveform^5,7,13^ CSTMD1 has a large receptive field with a sharp transition at the visual midline, with targets traversing the contralateral hemisphere with respect to our recording location driving inhibition and the ipsilateral driving strong excitation. BSTMD1 has a large binocular receptive field, with a characteristic graded response in the contralateral hemisphere and a pure spiking response in the ipsilateral hemisphere. BSTMD2 also has a large binocular receptive field, but it exhibits strong direction selectivity, with excitation for leftward moving targets, and inhibition for rightward moving targets in the ipsilateral visual field (recorded from the left lobula complex). Intracellular responses were digitized at 5 kHz with a 16-bit A/D converter (National Instruments) for off-line analysis with MATLAB.

The anatomy of all three STMD neurons presented here have been published elsewhere^5,7,13^, but we will briefly summarise them here. All three STMD neurons receive input in the lateral midbrain, and in the case of BSTMD1 also the medial lobula. CSTMD1 and BSTMD2 have large output arborisations which extend into the outer layers of the lobula, while the outputs of BSTMD1 are confined within the midbrain. Our previous work hypothesised synaptic interactions between CSTMD1 and BSTMD1, but evidence of this remains at a preliminary stage, and interactions with the more recently described BSTMD2 have not yet been investigated. All three neurons have arborisations in similar regions of the lateral midbrain and significant regions of overlap in their receptive fields, but at this time we lack convincing empirical evidence of direct synaptic interactions.

We sampled many neurons during our recordings, however present both individual exemplars and aggregate data. Precise spike timing varies between animals and pooling across animals smears inter-spike intervals (ISI). We performed this analysis on multiple examples of each neuron type and observed the same result. Therefore, we present visualisation of individual representative neurons and aggregate analysis of burst coding’s effect on response probabilities.

### Visual stimulation

**Figure 1:**
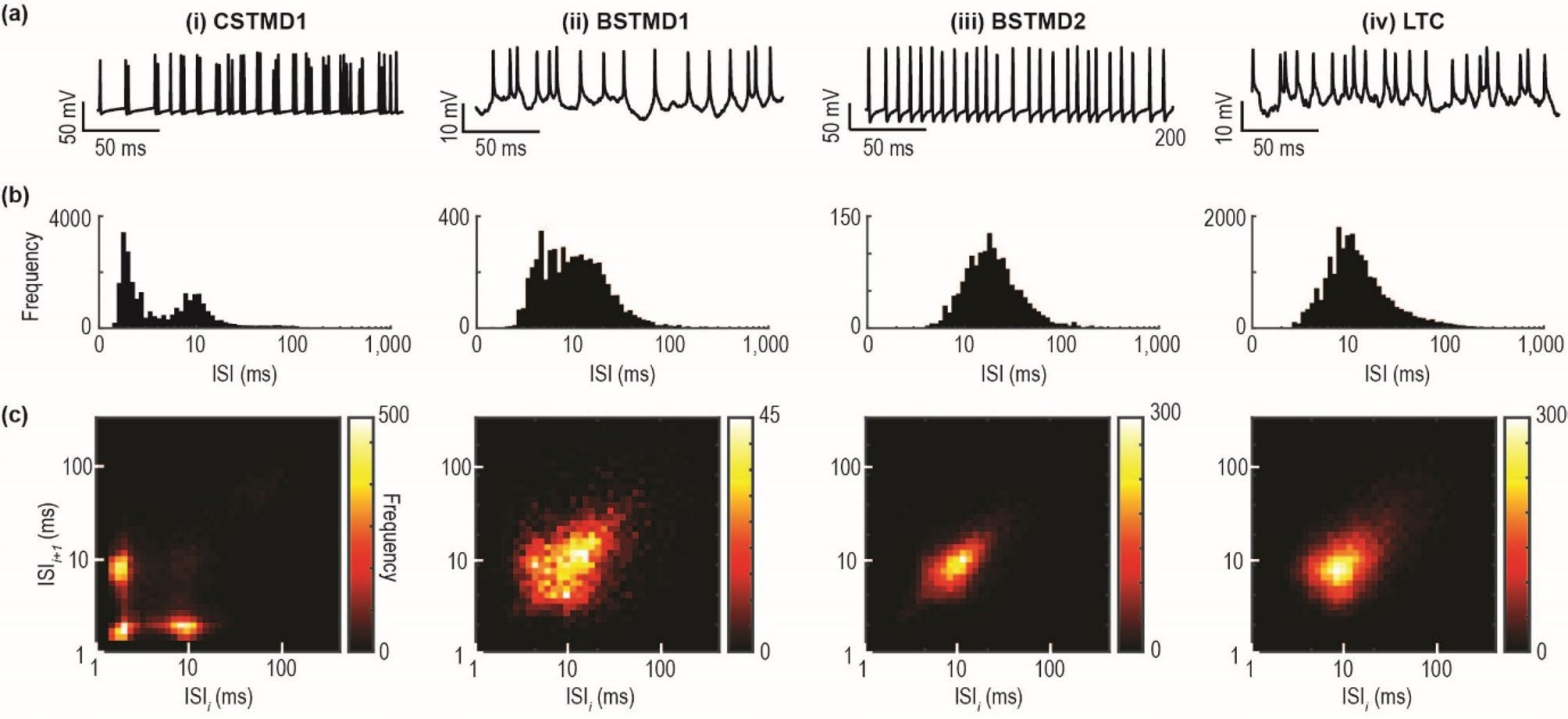
Statistics of spiking activity in dragonfly visual neurons. (**a**) Raw intracellularly recorded spike trains from 4 different dragonfly visual neurons during the presentation of a visual stimulus. (**b**) The distribution of ISI values from an individual example of each visual neuron. (**c**) The next interval (ISI_i+1_) plotted as a function of the previous interval (ISI_i_) for each neuron.

**Figure 2:**
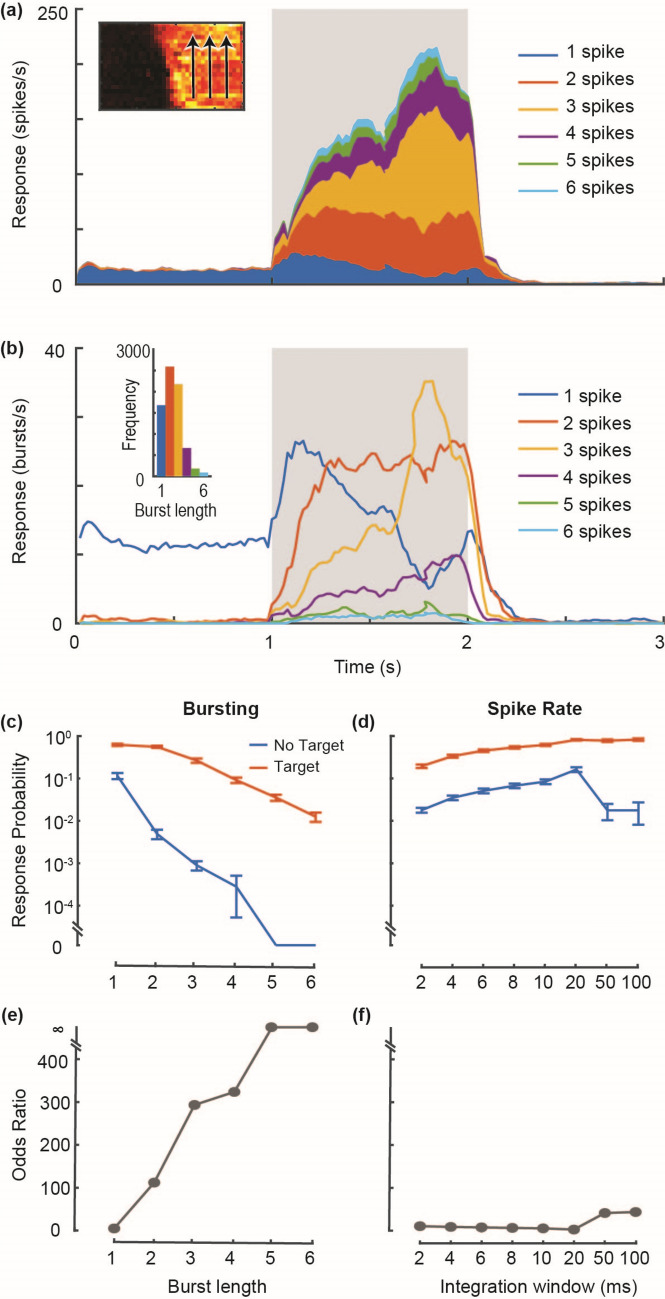
CSTMD1 responses segmented into component burst lengths. (**a**) An individual CSTMD1’s overall spiking activity in response to a drifting target (mean of 120 repetitions split between 3 possible trajectories in 1 dragonfly), is segmented into contributions from each burst length. Gray shaded region indicates peristimulus duration, and the inset shows the three target trajectories superimposed on CSTMD1s receptive field. (**b**) The same data is represented as the frequency rate of each burst length. The frequency of each burst length during target presentation (shaded region) is shown in the inset. (c) The probability of observing each burst length (20 ms windows), either in the presence (blue line) or absence (orange line) of a target drifting through CSTMD1’s receptive field (mean ± SEM, N = 17 dragonflies). (**d**) The probability of observing spike rates above 50 spikes/s in the presence or absence of a target, for a range of integration window sizes, for the same data as (**c**). (**e**,**f**) The odds ratios for bursts and spike rates, as calculated from the data in c and d.

We present visual stimuli on an LCD display (144–165 Hz) located 20 cm from the dragonfly, centred on the visual midline. We used custom software in MATLAB (utilising Psychtoolbox) to generate visual stimuli and synchronise data acquisition. Once identified, STMD neurons were presented with moving black square targets (1.5–2 deg) drifting at 60 degrees/s on a white background. Stimuli in Fig. 1(ii,iii) and Fig. 2c–f drifted at 21 different trajectories through the receptive field, while stimuli in Figs. 1(i) and 2a,b drifted vertically on 3 different trajectories. For Fig. 1(iv) LTCs were presented with elongated bars drifting in the preferred direction at a range of velocities and widths.

### Spiking and bursting analysis

We detect spike timing from intracellularly recorded signals with custom MATLAB scripts, and calculate interspike intervals (ISI). To visualise higher-order patterns in spike timing, we calculate the interval following a spike (ISI_*i*_) as a function of the preceding interval (ISI_*i*−1_). Bursts were defined as successive spikes with an ISI less than 5 ms. This threshold is consistent with other studies of bursting neurons^3^ and corresponded to a local minimum in CSTMD1’s ISI distribution. Burst length was defined as the number of consecutive spikes with ISI values below 5 ms.

To compute response probabilities, in the bursting condition we binned the data into 20 ms windows for all trials within an individual cell, and calculated the probability that any given window contained a burst of each length. In the spike rate condition, we binned the responses into a range of time windows, and calculated the probability that any given window reached a spike rate greater than 50 spikes/s $\left(spike\, rate= \frac{spike\, number}{window \,duration}\right)$. We define the odds ratio as the probability of a response (a burst or spike rate surpassing our threshold) occurring in the presence of a drifting target divided by the probability of a response in the absence of a target.

## Results

### Spiking statistics of dragonfly lobula neurons

Dragonfly visual neurons respond to a salient stimulus with a series of spikes (Fig. 1a). ISI is derived from the time separating any two successive pairs of spikes. The instantaneous spike rate can be calculated by inverting the ISI and will be similar to a spike rate determined by counting spikes within a ‘binned’ time window. Neuronal responses often have a log-normal distribution of ISIs, but in the case of CSTMD1 the skewed distribution reveals a second hump (Fig. 1b). This pattern of either extremely short ISIs, or relatively long ones, is indicative of a neuron which rapidly switches between very high and relatively low instantaneous spike rates. This is observed when neurons are firing spikes in bursts, with multiple action potentials occurring in brief succession, followed by a prolonged period of inactivity. The ISIs of the other neurons exhibit a broad log-normal distribution, representative of spike rates observed in previous studies and indicative of non-bursting neurons^7,13,14^.

In mammalian systems and the lateral line of weakly electric fish, burst coding neurons have been identified by comparing the ISIs between the previous spike and the next spike, for each spike in a train^1^. If a neuron spikes at a relatively constant rate, the ISI for the previous and the next spike should be similar, and when plotted against each other, points will cluster centrally. Conversely, if a neuron is exhibiting bursting behaviour, the first spike of a burst will have a large previous ISI and a small next ISI. The central spikes within a burst will have short ISIs in both directions. The final spike of a burst will have a small previous ISI and a large next ISI. Therefore, bursting neurons are identified by the formation of three separate clusters. This individual CSTMD1 exhibits a dominant bursting behaviour, in contrast to BSTMD1, BSTMD2 and this LTC which do not (Fig. 1c).

### Bursting behaviour in CSTMD1

CSTMD1 is differentiated by the bursting activity and we can compute the length of each burst, as well as the frequency of different burst lengths. We presented 120 repeats of a small (2 × 2 deg) target drifting vertically through the receptive field of CSTMD1 and computed the timing of each length of spike burst (e.g. single spike, 2 spike burst, etc.). We multiplied the frequency of each burst by the burst length to identify the proportion of total spikes which belong to each burst length across time (Fig. 2a). Spontaneous activity is almost exclusively composed of isolated spikes, but responses to a moving target are dominated by two and three spike bursts. The proportion of burst length vary across time, with higher burst lengths becoming more frequent as overall response strength increases. If we take the burst based view, bursts of different lengths follow different response time courses (Fig. 2b). During spontaneous activity and early stages of the target trajectory, almost all responses are isolated spikes. Over time as the target traverses into the most sensitive part of the receptive field spike activity increases, and burst length gradually increases with longer spike bursts becoming more common. The silent period following each burst lasts approximately 10 ms irrespective of the length of the burst, and the size of CSTMD1s receptive field gradually shrinks as spike burst length increases (see Supplemental Material).

Burst coding results in neuronal responses with higher signal to noise ratios (SNR) than spike rate codes in several systems^1^. For CSTMD1’s responses, we quantified the probability of each burst length occurring (across 17 dragonflies) in the presence, and absence (spontaneous activity) of a moving target. When no target is present isolated spikes are common and bursts are exceedingly rare, while bursts of 5 spikes or more were non-existent (Fig. 2c). When a target is moving within CSTMD1’s receptive field, the probability of all bursts increased. CSTMD1s responses are normally quantified by computing a spike rate over an integration window in time. Spike bursts can be detected very rapidly, for example if we assume a 2 ms ISI for each spike in a burst then a 5 spike burst is measurable within 8 ms of the first spike. For comparison we compute spike rates for the same data over a range of windows between 2 and 100 ms, and calculate the probability that those spike rates pass a threshold of 50 spikes/s (Fig. 2d). The probability of breaking this threshold was elevated when a target is present across all integration windows and increases with longer integration windows. In the absence of a target, long integration windows have reduced response probabilities, but the probability of these ‘false positives’ increases at shorter window lengths. From this data we can compute odds ratios for responses in the presence and absence of a target (N = 17). Doing so reveals that CSTMD1’s spike bursts (Fig. 2e) greatly outperform spike rates even at the longest integration windows tested (Fig. 2f). Robust spike rate calculation requires lengthy integration windows, but the presence of a burst only takes a few milliseconds, highlighting bursting as an excellent coding solution for signalling robust, rapid signals.

## Discussion

Here we have provided strong evidence of bursting activity in a well-studied target-detecting neuron in the dragonfly brain. To our knowledge, this is the first example of this form of spiking activity in any insect target-detecting neuron. Bursting activity conveys multiple potential advantages when compared to a rate based alternative. Spike bursting increases the reliability of synaptic transmission by increasing redundancy, which may be important given the relatively low probabilities of vesicle release from individual spike arrival at many synapses^15^. In this sense, using spike bursts increases the information content of a spike train at the cost of temporal resolution and metabolic energy. In addition to this, synapses could be tuned to selectively detect subsets of burst length, allowing the parallel coding and decoding of multiple signals from a single spike train^16^.

How might CSTMD1 use spike bursts? We have demonstrated that the other two identified STMD neurons, BSTMD1 and BSTMD2, do not exhibit bursting activity, so this is not a common property of all STMD neurons. Moving targets elicit a range of burst lengths, and we are yet to identify a stimulus parameter which selectively drives specific bursts. Further work should focus on varying target parameters such as size or velocity, to determine whether tuning curves differ between varying burst lengths. Spike bursts provide a more robust indicator of target movement than isolated spikes. CSTMD1 is spontaneously active, firing 10–20 spikes each second in the absence of visual stimulation. Downstream neurons cannot distinguish spontaneous from stimulus driven spikes, therefore any individual spike encodes relatively little information about target movement. However, spike bursts are extremely rare in the absence of a stimulus, and the likelihood of bursts decreases with increasing length. This means that spike bursts represent a coding space which is almost completely free of background noise, and could be used to signal critical events to downstream neurons which require higher levels of confidence.

Using spike rates to encode information comes with many limitations, evidenced both with empirical and theoretical study^17^. Neuronal responses are usually reported as spike rates because they are convenient to measure and correlated with information content, however in many circuits, it is not clear whether they play a causal role in neuronal communication. If CSTMD1 encodes information by spike rate, a downstream neuron must integrate spiking activity over a window of time to estimate that rate. In such systems, shorter integration times results in larger quantisation errors in spike rate estimation, so robust signals require long integration windows. Dragonfly prey pursuits involve rapidly changing conditions, and behaviour is controlled with minimal latency^18,19^. Our data shows that short integration windows for spike rate calculation leads to an uninformative signal, and integration times of 50 ms or more are required to see significant improvement. Spike bursting is an extremely effective method for signalling information rapidly and accurately, since spike bursts have durations of only a few milliseconds, and occur almost exclusively during the presentation of a relevant stimulus. In fact all spike bursts had odds ratio greater than even the longest spike rate integration times tested.

If spike bursting has so many benefits for target detection, why don’t all target detecting neurons burst? The answer to this question remains elusive given our current understanding of STMD circuits, but it may be linked unique properties of CSTMD1. Bursting systems rely on interactions between rapid spike generation conductances and slower mechanisms which determine when these bursts occur^1^. CSTMD1 is known for its strong facilitative mechanisms, where responses gradually build as a target drifts through the receptive field^6,7,10,11^, and it is possible that mechanisms underlying bursting and facilitation are linked. While there is still evidence of facilitatory mechanisms in BSTMD1 and BSTMD2^10^, the strength of this facilitation tends to be far weaker than what we observe in CSTMD1. Secondly, many of the advantages of busting relies on the ability of downstream neurons to decode these bursts. Very little is known about the downstream targets of specific STMD neurons, and until these circuits are identified the functional implications of bursting, or the absence of bursting, will be elusive. Further investigation will examine how CSTMD1′s gradual increase of bursts and decrease in isolated spikes relates to the slower mechanisms driving facilitation. Moreover, it is important to establish how this bursting code may be intertwined with the previously observed ‘higher-order’ properties of prediction or selective attention.

## Supplementary Information

Supplementary Information.

## Data Availability

The datasets generated during the current study are available in the Figshare repository (https://doi.org/10.25909/13277879).
