# Author Correction: Spike bursting in a dragonfly target-detecting neuron

**DOI:** 10.1038/s41598-021-89240-1

**Published:** 2021-04-29

**Authors:** Joseph M. Fabian, Steven D. Wiederman

**Affiliations:** 1 Centre for Neuroscience, Flinders University, Adelaide, SA Australia; 2 Adelaide Medical School, The University of Adelaide, Adelaide, SA 5005 Australia

Correction to: *Scientific Reports* https://doi.org/10.1038/s41598-021-83559-5, published online 17 February 2021

This Article contains errors in Reference 13 which is incorrectly given as:

Evans, B. J. E., Fabian, J. M., O’Carroll, D. C. & Wiederman, S. D. Dragonfly visual neurons selectively attend to features in naturalistic scenes. J. Neurosci. 39, 8051 (2020).

The correct Reference 13 appears below as Reference^1^.
