# Supplementary material for: Spike bursting in a dragonfly target-detecting neuron
Source: Sci Rep. 2021 Feb 17;11:4005. doi: 10.1038/s41598-021-83559-5 (PMC7889644; doi:10.1038/s41598-021-83559-5)
Supplement: Supplementary file 1 — Supplementary Information. [file 41598_2021_83559_MOESM1_ESM.docx]

**Supplemental Information for: Spike bursting in a dragonfly target-detecting neuron**

Joseph M. Fabian^1^ & Steven D. Wiederman^2^

1. Centre for Neuroscience, Flinders University, Adelaide, SA
2. Adelaide Medical School, The University of Adelaide, Adelaide, SA 5005


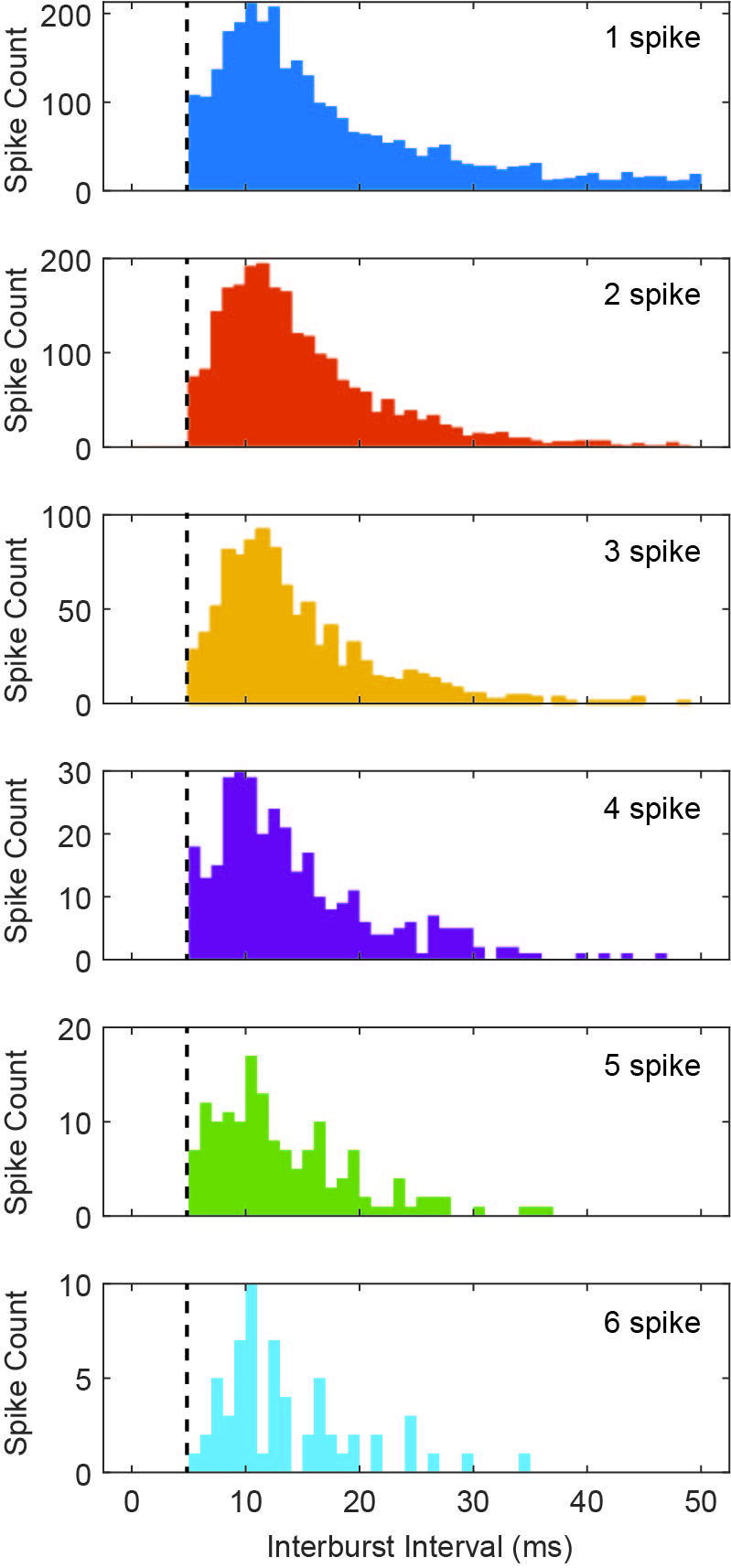


**Supplemental figure S1: Interburst intervals for each burst length in CSTMD1**

We computed the interval between the last spike in each burst length (1-6 spikes) and the next spike following the burst, for the same data in figure 2a-b. Dashed line indicates the threshold for burst detection, so by definition no interburst intervals can fall below this threshold.


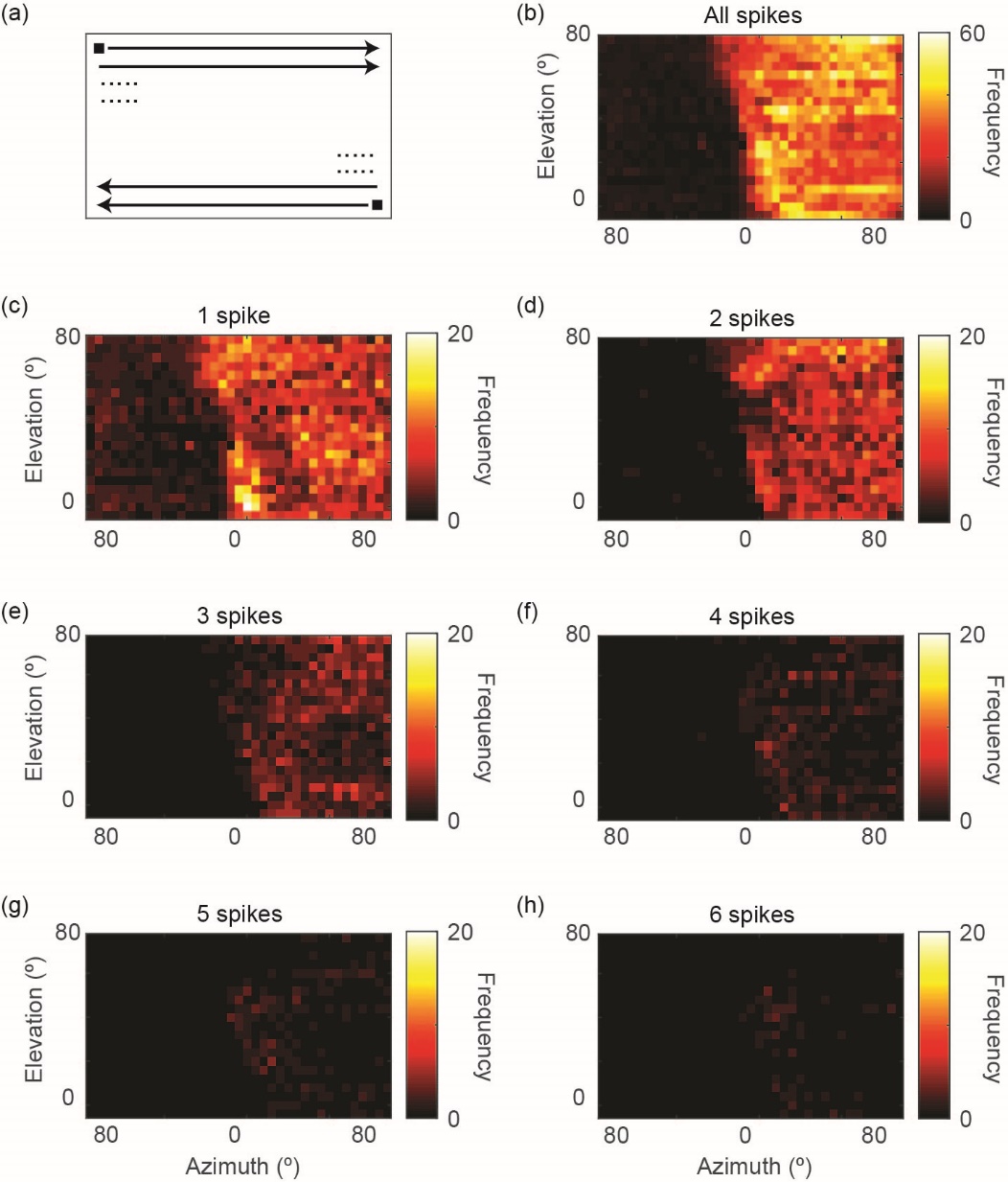


**Supplemental figure S2: The receptive field of CSTMD1, for each burst length.**

(a) The receptive field of an individual CSTMD1 neuron is scanned by a series of targets drifting horizontally across the visual display in both directions. (b) The receptive field displaying all spikes, irrespective of any bursting activity. (c-h) The receptive field of the same cell, split into each bursting component.
